# Improved Complete Genome Sequence of the Extremely Radioresistant Bacterium *Deinococcus radiodurans* R1 Obtained Using PacBio Single-Molecule Sequencing

**DOI:** 10.1128/genomeA.00886-16

**Published:** 2016-09-01

**Authors:** Xiaoting Hua, Yuejin Hua

**Affiliations:** a Key Laboratory of Chinese Ministry of Agriculture for Nuclear-Agricultural Sciences, Institute of Nuclear-Agricultural Sciences, Zhejiang University, Hangzhou, Zhejiang, People's Republic of China; b Department of Infectious Diseases, Sir Run Run Shaw Hospital, College of Medicine, Zhejiang University, Hangzhou, Zhejiang, People's Republic of China

## Abstract

The genome sequence of *Deinococcus radiodurans* R1 was published in 1999. We resequenced *D. radiodurans* R1 using PacBio and compared the sequence with the published one. Large insertions and single nucleotide polymorphisms (SNPs) were observed among the genome sequences. A more accurate genome sequence will be helpful to studies of *D. radiodurans.*

## GENOME ANNOUNCEMENT

*Deinococcus radiodurans* R1 is extremely resistant to the lethal effects of ionizing radiation (IR), UV light, oxidation, and desiccation (1). It can survive doses of ionizing radiation of >12,000 Gy, 3,000 times higher than for most vertebrates (2). Different resistance mechanisms have been proposed to explain the extreme radioresistance of the bacterium after its discovery in the 1950s (3). Efficient scavenging of reactive oxygen species and repair of damaged DNA were considered as two of these (4). Over the past decade, many genetic, biochemical, biophysical, and structural studies focused on the DNA repair mechanism of *D. radiodurans* (5–8). These studies were based on the reference sequence of *D. radiodurans*. However, the current genome sequence of *D. radiodurans* R1 was finished in 1999, and contained a number of mistakes in the sequence (9). To fully facilitate studies of *D. radioduran*, it was necessary to resequence the genome of *D. radiodurans* R1 to provide more accuracy and higher quality.

Here, we present the complete genome sequence of *D. radiodurans* R1, obtained using Pacific BioSciences (PacBio) sequencing technology. Genomic DNA of strain R1 was extracted using a QIAamp DNA minikit (Qiagen, Valencia, CA) following the protocol of the manufacturer. The quality of DNA was determined by gel electrophoresis and a NanoDrop 2000 spectrophotometer (Nano-drop Technologies, Wilmington, DE). After the library construction, the genome was sequenced by the PacBio RS platform. A total of 59,327 polymerase reads with a mean read length of 11,445 bases were generated, which led to a total of 679,027,015 bases with a 176-fold average coverage. *De novo* assembly of the read sequences was performed using continuous long reads following the Hierarchical Genome Assembly Process (HGAP) workflow (PacBio DevNet; Pacific Biosciences) as available in SMRT Analysis v2.3.0. Annotation of *D. radiodurans* R1 was performed using the NCBI PGAAP annotation pipeline and manually checked. The pipeline uses Genemark to predict open reading frames (ORF) and searches against Proteins Clusters. Protein coding genes were searched against the NCBI RefSeq database using BLASTp.

The genome of *D. radiodurans* R1 is 3,344,765 nucleotides, 66.3% G+C content, and contains two circular chromosomes and two circular plasmids. Among of the 3,212 genes predicted, 3,079 were protein-coding genes. Sixty-two RNAs were also identified. Comparative genome analysis of the genome sequence from this study and the published one was performed with Mauve (10). Large insertions were observed in two chromosomes and two plasmids. Moreover, small insertions and deletions frequently happened in the genome sequence of the bacterium. After mapping the raw sequence data to the previous genome sequence, 92 deletions, 297 insertions, and 188 substitutions were observed. For the DNA repair related gene, frameshifts were detected in the *ssb* gene, which confirmed a previous report. The genome sequence presented here will be helpful for elucidating the radioresistance mechanism in *D. radiodurans*.

### Accession number(s).

The sequence data for the genome of *D. radiodurans* R1 have been deposited in GenBank under accession numbers CP015081 to CP015084.

## References

[1] Byrne RT, Klingele AJ, Cabot EL, Schackwitz WS, Martin JA, Martin J, Wang Z, Wood EA, Pennacchio C, Pennacchio LA, Perna NT, Battista JR, Cox MM 2014 Evolution of extreme resistance to ionizing radiation via genetic adaptation of DNA repair. Elife 3:e01322. doi:10.7554/eLife.01322. 24596148 PMC3939492

[2] Tsai CH, Liao R, Chou B, Contreras LM 2015 Transcriptional analysis of *Deinococcus radiodurans* reveals novel small RNAs that are differentially expressed under ionizing radiation. Appl Environ Microbiol 81:1754–1764. doi:10.1128/AEM.03709-14. 25548054 PMC4325154

[3] Pavlopoulou A, Savva GD, Louka M, Bagos PG, Vorgias CE, Michalopoulos I, Georgakilas AG 2016 Unraveling the mechanisms of extreme radioresistance in prokaryotes: lessons from nature. Mutat Res Rev Mutat Res 767:92–107. doi:10.1016/j.mrrev.2015.10.001. 27036069

[4] Slade D, Radman M 2011 Oxidative stress resistance in *Deinococcus radiodurans*. Microbiol Mol Biol Rev 75:133–191. doi:10.1128/MMBR.00015-10. 21372322 PMC3063356

[5] Cheng K, Xu H, Chen X, Wang L, Tian B, Zhao Y, Hua Y 2016 Structural basis for DNA 5′-end resection by RecJ. Elife 5:e14294. doi:10.7554/eLife.14294. 27058167 PMC4846377

[6] Sugiman-Marangos SN, Weiss YM, Junop MS 2016 Mechanism for accurate, protein-assisted DNA annealing by *Deinococcus radiodurans* DdrB. Proc Natl Acad Sci USA 113:4308–4313. doi:10.1073/pnas.1520847113. 27044084 PMC4843489

[7] Jeong SW, Jung JH, Kim MK, Seo HS, Lim HM, Lim S 2016 The three catalases in *Deinococcus radiodurans*: only two show catalase activity. Biochem Biophys Res Commun 469:443–448. doi:10.1016/j.bbrc.2015.12.017. 26692481

[8] Ithurbide S, Bentchikou E, Coste G, Bost B, Servant P, Sommer S 2015 Single strand annealing plays a major role in RecA-independent recombination between repeated sequences in the radioresistant *Deinococcus radiodurans* bacterium. PLoS Genet 11:e1005636. doi:10.1371/journal.pgen.1005636. 26517555 PMC4627823

[9] White O, Eisen JA, Heidelberg JF, Hickey EK, Peterson JD, Dodson RJ, Haft DH, Gwinn ML, Nelson WC, Richardson DL, Moffat KS, Qin H, Jiang L, Pamphile W, Crosby M, Shen M, Vamathevan JJ, Lam P, McDonald L, Utterback T, Zalewski C, Makarova KS, Aravind L, Daly MJ, Minton KW, Fleischmann RD, Ketchum KA, Nelson KE, Salzberg S, Smith HO, Venter JC, Fraser CM 1999 Genome sequence of the radioresistant bacterium *Deinococcus radiodurans* R1. Science 286:1571–1577. doi:10.1126/science.286.5444.1571. 10567266 PMC4147723

[10] Darling AC, Mau B, Blattner FR, Perna NT 2004 Mauve: multiple alignment of conserved genomic sequence with rearrangements. Genome Res 14:1394–1403. doi:10.1101/gr.2289704. 15231754 PMC442156

